# Prevalence and Seroprevalence of Infectious Bronchitis Virus and Infectious Laryngotracheitis Virus in Backyard Poultry in Central Chile

**DOI:** 10.3390/ani15162364

**Published:** 2025-08-12

**Authors:** Cecilia Baumberger, Francisca Di Pillo, David Tapia, Claudio Coloma, Katherinne Orozco, Pablo Galdames, Cristobal Oyarzun, Diego Gárate, Camila Torreblanca, Soledad Ruiz, Pedro Jimenez-Bluhm, Christopher Hamilton-West

**Affiliations:** 1 Departamento de Medicina Preventiva Animal, Facultad de Ciencias Veterinarias y Pecuarias, Universidad de Chile, Santiago 8820808, Chile; 2 Núcleo de Investigación en One Health, Facultad de Medicina Veterinaria y Agronomía, Universidad de Las Américas, Providencia, Santiago 7500590, Chile; 3 Escuela de Medicina Veterinaria, Facultad de Recursos Naturales y Medicina Veterinaria, Universidad Santo Tomás, Santiago 8370003, Chile; 4 Escuela de Medicina Veterinaria, Facultad de Medicina, Facultad de Ciencias Biológicas y Facultad de Agronomía y Sistemas Naturales, Pontificia Universidad Católica de Chile, Santiago 7820436, Chile

**Keywords:** avian pathogens, IBV, ILTV, backyard poultry, avian diseases, seroprevalence, surveillance, Chile

## Abstract

Backyard production systems (BPSs) are common in Chile and play an important role in food access and local trade. However, these systems often lack basic biosecurity and disease prevention practices, which increases the risk of disease spreading. In this study, we evaluated the presence of two major avian respiratory viruses, infectious bronchitis virus (IBV) and infectious laryngotracheitis virus (ILTV), in BPSs located near wetlands in central Chile. These areas are known as the country’s main poultry production zones. We collected 449 poultry serum samples from 88 BPSs and performed serological tests using ELISA. Additionally, we analyzed 250 poultry tracheal swabs from 31 BPSs using qPCR. The results showed high seroprevalence levels: 95.5% of BPSs tested positive for IBV and 85.2% for ILTV. At the animal level, 82.2% were positive for IBV and 57.2% for ILTV. Most birds had antibodies to both viruses. However, active infections were less frequent, with 4.3% of tracheal swabs testing positive for IBV and 14.1% for ILTV during 2021 and 0.6% and 3.8% for IBV and ILTV, respectively, during 2024. This is the first serological and molecular evidence of IBV and ILTV circulation in backyard poultry in central Chile. Since this region includes most of the country’s poultry industry, these findings raise concern about the risk of virus transmission to commercial farms. The high circulation rates suggest that backyard poultry could act as reservoirs and may contribute to decreased productivity. Our results highlight the need for improved disease surveillance and enhancement of biosecurity in BPSs in Chile.

## Simple Summary

Backyard poultry systems are small-scale farms commonly found in rural areas, where families raise chickens and other birds mainly for food and local trade. These farms often lack basic disease prevention measures, which makes it easier for infections to spread. In this study, we tested birds from 88 backyard farms in central Chile. We investigated two important respiratory diseases: avian infectious bronchitis and avian infectious laryngotracheitis. These diseases can reduce egg production, slow bird growth, and cause illness or death. We found that most birds had been exposed to one or both viruses. However, fewer birds had active infections at the time of sampling. The widespread presence of these viruses is concerning because this region is also where most of Chile’s commercial poultry farms are located. The results suggest that backyard poultry could act as reservoirs of disease, putting both small-scale and large-scale poultry production at risk. Improving disease prevention and awareness in backyard farms is crucial to protecting animal health, ensuring a stable food supply, and maintaining family livelihoods.

## 1. Introduction

Poultry production has experienced remarkable growth within the livestock industry, becoming a favored choice for animal protein worldwide, including both meat and eggs [1]. Economic and market projections indicate an increase in global meat protein consumption by 2030, with poultry expected to represent 41% of all meat sources globally [2]. While high-density and fully commercial broiler systems contribute significantly to the sector’s growth and global production, it is essential to acknowledge the continued significance of small-scale poultry production [3]. These traditional systems remain important in numerous developing and industrialized countries [4,5,6]. Within small-scale production settings, backyard production systems (BPSs) are recognized as the most widely distributed form of animal production in the world [3].

Backyards are characterized by rearing multiple poultry species in variable flock sizes, with household consumption and informal trade as the primary destinations for poultry products, thereby providing an economic contribution to low-income families, mainly in rural areas [7,8]. Nevertheless, backyards usually lack the traditional biosecurity measures commonly observed in highly industrialized production systems, making them more susceptible to the introduction, maintenance, and dissemination of infectious pathogens [7,9,10,11]. Additionally, these systems act as an interface where domestic poultry species interact with wild animals, as domestic poultry kept in BPSs frequently roam freely in search of food [7,10,11,12]. The interface between backyard poultry and wild birds is recognized as a high-risk area for the movement of priority pathogens affecting the poultry industry, as well as pathogens with zoonotic risk [13]. This phenomenon has been previously documented in Chile, where systematic surveillance studies of avian influenza virus have revealed spillover from wild birds to backyard poultry [10]. Consequently, BPSs may serve as reservoirs and amplifiers of infectious diseases, significantly impacting both the commercial poultry industry and public health. Respiratory pathogens have a direct economic impact, primarily through increased animal mortality and decreased productivity indicators. Indirect costs are also incurred, encompassing treatments, vaccines, surveillance, as well as market losses due to restrictions on international trade [14,15].

Infectious bronchitis virus (IBV) and infectious laryngotracheitis virus (ILTV) are among the respiratory tract pathogens with the most significant economic impact [16,17]. Birds infected with these pathogens exhibit a range of clinical signs, including cough, respiratory distress, conjunctivitis, sinusitis, ocular and nasal discharge, general fatigue, reduced feed intake, increased morbidity, and substantial mortality [18,19,20,21,22]. Furthermore, laying hens undergo a decline in both egg production and quality. Moreover, these pathogens can induce co-infections or multiple infections, potentially involving other viral, bacterial, or fungal pathogens, as sick birds tend to be more susceptible to secondary respiratory tract infections [23,24].

In Chile, poultry meat constitutes the primary form of meat production and consumption. Throughout 2021, the poultry industry made substantial contributions to both the country’s economy and food security, accounting for 48% of the total livestock production, with a significant proportion allocated to export [25]. Based on the last national agricultural census, BPSs account for 3.4% of the national poultry stock, with a concentration mainly in central Chile [26]. Despite the endemic nature of IBV and ILTV in Chile, there exists limited knowledge regarding the prevalence of these pathogens in both industrial and backyard poultry systems. Prior research has primarily focused on characterizing IBV isolates from the poultry industry [27,28,29]; however, minimal attention has been given to the study of ILTV, except for a few instances concerning wild bird reports [30] and one study describing its circulation in BPSs in the southern region of Chile [31]. Therefore, the objective of this study was to investigate the prevalence and seroprevalence of IBV and ILTV in backyard poultry in central Chile.

## 2. Materials and Methods

### 2.1. Study Area and Study Design

The central region of the country has a Mediterranean climate [32], which has proven advantageous for both agricultural and animal husbandry activities [33]. Consequently, around 95% of poultry production is concentrated in central Chile, involving the rearing of over 43.5 million birds, which accounts for a substantial 83% of the total national poultry population. This same geographical region also hosts a significant number of BPSs dedicated to poultry rearing [8], which have been designated as the study units in this study.

**Figure 1:**
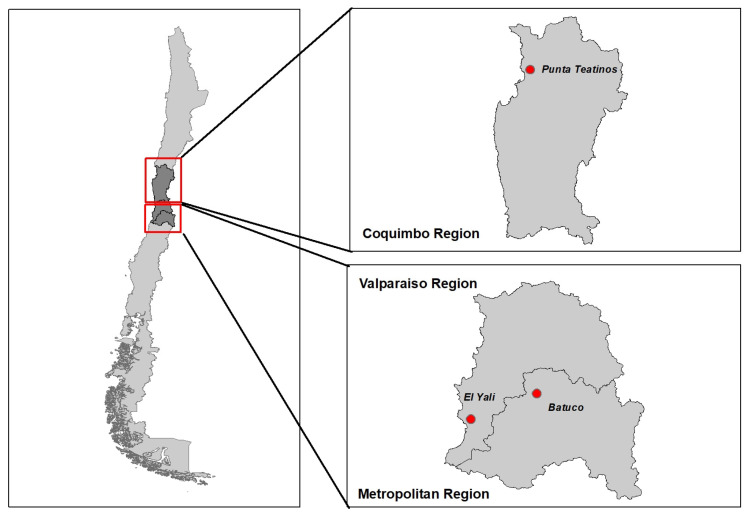
The study areas comprising poultry backyards in the proximity of Batuco wetland in the Metropolitan region, El Yali National Reserve in the Valparaiso region, and Punta Teatinos in the Coquimbo region.

The study areas were defined based on wetlands recognized as important wild aquatic bird concentration areas in central Chile: (i) Punta Teatinos (Coquimbo region), (ii) Batuco wetland (Metropolitan region), and (iii) El Yali National Reserve (Valparaíso region) (Figure 1). The target population included poultry BPSs located in the proximity of these wetlands. A cross-sectional study was undertaken of a total of 88 poultry BPSs that were visited during January and February 2021. A BPS was defined as a family farm unit breeding up to 100 birds [8]. Blood samples were collected from eight birds per BPS, or the entire flock if the BPS had fewer than eight birds. Blood samples were obtained from the brachial vein (1–3 mL) using blood collection tubes and refrigerated at 4 °C upon arrival at the laboratory at the Faculty of Veterinary Medicine, University of Chile. Serum was obtained by centrifugation at 1300× *g* for 15 min and stored at −20 °C until analysis. Parallel to this, tracheal swabs were obtained from 92 birds from 11 BPSs from Batuco and El Yali National Reserve study areas. The COVID-19 pandemic led to the early termination of the first sampling season due to movement restrictions; therefore, a second season was undertaken in November 2024 to increase the sample size for estimating prevalence. The second sampling season included 158 tracheal swabs collected from poultry belonging to 20 BPSs located in Batuco and El Yali National Reserve. Disposable sterile swabs were placed into vials containing 1 mL of Universal Transport Media for sampling (Copan Group, Brescia, BS, Italy). Swab samples were kept at 4 °C during transportation and stored at −80 °C upon arrival at the laboratory.

### 2.2. Laboratory Analysis

Commercial ELISA kits were employed for serological analysis to determine the seroprevalence of IBV (CK119) and ILT (CK124; BioChek BV, Reeuwijk, The Netherlands), following the manufacturer’s instructions. Each serum sample was tested in a single well of the antigen-coated plates provided in the kits for each pathogen. In brief, 100 µL of diluted serum (1:500) was added to the well and incubated for 30 min (IBV) or 1 h (ILTV) at room temperature. Subsequently, each well was washed four times with 350 µL of wash buffer, and 100 µL of conjugate reagent was added to the wells. After a second incubation at room temperature, plates were washed as described above. Following this, 100 µL of substrate reagent was added to each well, and the plate was incubated for an additional 15 min. Finally, the reaction in each well was quenched with 100 µL of stop solution. Absorbance was read using a Sunrise TM microplate reader (Tecan Group AG, Männedorf, Switzerland). Positive and negative controls provided in the kits were included in all plates. To estimate prevalence, tracheal swabs were analyzed using qPCR. Nucleic acid extraction from swab samples was performed using a MagMax AI/ND-96 viral extraction kit (AM1835; ThermoFisher Scientific, MA, USA). For qPCR, commercial kits were used for IBV (CP108) and ILTV (CP104; BioChek BV, Reeuwijk, The Netherlands) on a Mx3000P Stratagene™ thermocycler (Agilent Technologies, CA, USA), following the manufacturer’s instructions. Reactions were run according to the manufacturer’s recommended cycling conditions. For IBV detection (RNA virus), the qPCR program included an initial reverse transcription step at 48 °C for 10 min, followed by 95 °C for 3 min and 40 cycles of 95 °C for 15 s and 60 °C for 60 s. For ILTV detection (DNA virus), the protocol started directly at 95 °C for 3 min, followed by 40 cycles of 95 °C for 15 s and 60 °C for 60 s. Fluorescence data were collected during the 60 °C step. In both assays, the FAM channel was used for target detection (IBV or ILTV) and the Cy5 channel (Quasar 670) for the internal control. To ensure assay accuracy and reliability, each run included the kit-supplied controls: a negative control (molecular biology-grade water) and a positive control (plasmid with a cloned target sequence representing either IBV or ILTV).

### 2.3. Data Analysis

IBV and ILTV prevalence and seroprevalence and 95% confidence intervals were estimated overall and stratified by study area (seroprevalence) or sampling season (prevalence) using the normal approximation method [34]. A BPS was considered positive if at least one sample tested positive. The comparison of flock size by study area was performed using a Kruskal–Wallis test, and statistical significance was set at ≤0.05.

## 3. Results

### 3.1. Serological Surveillance

Across all study areas, a total of 449 serum samples (*n* = 229, *n* = 120, and *n* = 100 from Punta Teatinos, El Yali National Reserve, and Batuco, respectively) were collected from backyard poultry belonging to 88 BPSs. Median flock size was 40 birds (Q1 = 25; Q3 = 70) and did not differ for study areas (*p* = 0.783). Flocks were composed mainly of chickens; however, other poultry species (such as ducks or geese) were also present in 31% of BPSs. Seropositivity at the animal level was 82.2% (95% CI = 78.6–85.7%) and 57.2% (95% CI = 52.7–61.8%) for IBV and ILTV, respectively. Most of the birds (51.2%) tested seropositive for both pathogens, while only 53 birds (11.8%) were seronegative for both. Overall seroprevalence at the BPS level was 95.5% (95% CI = 91.1–99.8%) and 85.2% (95% CI = 77.8–92.6%) for IBV and ILTV, respectively. As shown in Table 1, seroprevalence at the BPS level among study areas ranged between 93.1% and 100.0% for IBV and between 82.8% and 89.7% for ILTV (Table 1). Among IBV-seropositive BPSs, all screened poultry were seropositive in 71% of cases, compared to 36% for ILTV.

### 3.2. Molecular Surveillance

During the first sampling season, 92 tracheal swabs were collected from chickens, of which four (4.3%) tested qPCR-positive for IBV and 13 (14.1%) for ILTV. IBV-positive samples corresponded to three BPSs (27.3%) and ILTV-positive samples to eight BPSs (72.7%). During the second sampling season, 158 tracheal swab samples were collected from chickens, and 0.6% (belonging to one positive farm from 20 BPS; 5.0%) tested qPCR-positive for IBV and 3.8% (six positive samples belonging to five farms from 20 BPS; 25.0%) for ILTV (Table 2). No poultry tested qPCR-positive for both pathogens for either sampling season.

## 4. Discussion

This study assessed the prevalence and seroprevalence of IBV and ILTV in backyard poultry in central Chile. The results showed a significant circulation of both pathogens at the backyard level, constituting the first study investigating these viruses in such production settings in central Chile. Studies conducted in Chile over the last decade have revealed extensive deficiencies in the implementation of biosecurity measures in backyards. These studies identified non-permanent poultry confinement, the absence of functional fences to prevent contact with neighboring animals, the contact between poultry and wild birds, the presence of neighboring backyard animals, and the absence of footbaths, among other biosecurity deficiencies [7,8,9,10,11,12]. Moreover, BPSs often lack poultry disease management, including quarantine measures and treatment for birds exhibiting clinical signs, as well as vaccination campaigns [8]. Consequently, BPSs are recognized as high-risk hotspots for the introduction, maintenance, and spread of infectious diseases, underscoring the need for preventive and surveillance programs [8,35,36,37]. In our study, in 71% of IBV-seropositive BPSs, all screened birds were seropositive, suggesting substantial within-flock exposure in the absence of preventive health measures [38]. Both IBV and ILTV are typically included in Chile’s commercial poultry vaccination programs. However, outbreaks remain frequent and commonly underdiagnosed in small-scale production systems where vaccination is absent, primarily due to the unavailability of vaccine doses for small flocks. The dissemination of vaccine virus cannot be ruled out as a contributing factor to outbreaks in small-scale farms, given recent molecular evidence of vaccine-related ILTV strains circulating in unvaccinated backyard poultry in southern Chile [31]. Further studies, including viral genotyping, are needed to assess whether circulating viruses are genetically related to vaccine strains.

Our study revealed significant seroprevalence levels in backyard poultry at the BPS level, with 95.5% and 85.2% for IBV and ILTV, respectively. Conversely, poultry tracheal swabs analyzed using qPCR showed lower positivity rates, which may be attributed to the cross-sectional design employed in this study. Sampling was performed at a single time point at each BPS, potentially underestimating the number of infection events within the study population. The ILTV positivity rate at the animal level reported in this study (14.2% and 3.8% for 2021 and 2024, respectively) is lower compared to a recent study conducted in backyard poultry from southern Chile, which reported a positivity rate of 90% [31]. This difference could be attributed to the targeted population of each study. While Gatica and collaborators (2025) [31] evaluated upper respiratory tract samples obtained during necropsy of poultry exhibiting respiratory clinical signs, we evaluated tracheal swabs collected from asymptomatic poultry. Additionally, sample collection had to be interrupted due to the onset of the COVID-19 pandemic. Therefore, fewer BPSs were included in 2021 compared to the second sampling season, resulting in a lack of statistical power to detect differences in ILTV and IBV circulation between seasons. Further longitudinal studies are needed to better characterize transmission dynamics and seasonality. Results from the present study evidence a higher seroprevalence compared to previous cross-sectional studies conducted in poultry backyards [39,40]. This difference can be partially explained by the backyard population selected in our study, which was represented by farms with poor biosecurity implementation, located in proximity to wetlands. Consequently, a higher risk of contact between wild birds and backyard animals was expected. Verdugo and collaborators (2019) found a 20% prevalence of Gammacoronavirus closely related to IBV in Cormorants, which are one of the most abundant aquatic wild birds in Chile [30]. This adds to previous evidence of IBV in aquatic wild birds [41,42,43], indicating the potential role of wild birds as vectors facilitating the spread of IBV between wild and domestic avian populations [44].

Both IBV and ILTV significantly impact poultry production, leading to reduced egg production, altered egg quality, and decreased growth rates, resulting in substantial economic losses [18,38]. This is particularly relevant since only 62% of backyard farmers achieve a positive economic balance from poultry production, and the economic contribution (through household consumption or local trade) is especially important for backyards located further away from markets and for lower-income families [7]. Our findings suggest that both IBV and ILTV are circulating within poultry BPSs, potentially contributing to decreased productivity and negatively impacting overall poultry production outputs. The widespread detection of IBV and ILTV in BPSs in central Chile, where it had not been previously reported, is concerning, as this region concentrates most of the country’s poultry production and poses a potential risk for transmission to commercial farms. Enhanced surveillance, particularly through the use of molecular tests, is essential; however, these methods are often limited by cost and diagnostic turnaround time. Finally, the first sampling season of this study was conducted during the COVID-19 pandemic; therefore, interviews with backyard farmers were not included in this study. Further information on farm characteristics and animal management could have provided valuable insights into risk factors at the backyard level. Future research should explore these factors to develop evidence-based preventive measures.

## 5. Conclusions

This study provided the first serological and molecular evidence of IBV and ILTV in backyard poultry in central Chile. Our findings revealed high seroprevalence rates, indicating widespread circulation of these pathogens in BPSs. This evidence highlights the importance of enhanced molecular surveillance, particularly through qPCR, and the implementation of preventive measures in BPSs. Educational programs aimed at improving poultry disease signs among backyard farmers are essential for mitigating the negative impacts of respiratory infectious diseases.

## Figures and Tables

**Table 1:** Infectious bronchitis virus (IBV) and infectious laryngotracheitis virus (ILTV) seroprevalence at the backyard production system (BPS) level by study area (Batuco wetland, El Yali National Reserve, and Punta Teatinos) using ELISA.

Study Area	Pathogen	Seroprevalence (*n* (%))	Total BPSs (*n*)	Seroprevalence 95% CI (%)
Batuco	IBV	27 (93.1)	29	83.9–100.0
	ILTV	26 (89.7)	29	78.6–100.0
El Yali	IBV	30 (100.0)	30	88.4–100.0
	ILTV	25 (83.3)	30	70.0–96.7
Punta Teatinos	IBV	27 (93.1)	29	83.9–100.0
	ILTV	24 (82.8)	29	69.0–96.5

**Table 2:** Infectious bronchitis virus (IBV) and infectious laryngotracheitis virus (ILTV) prevalence at the backyard production system (BPS) level using qPCR.

Pathogen	Sampling Season	Prevalence (*n* (%))	Total BPSs (*n*)	Prevalence 95% CI (%)
IBV	2021	3 (27.3)	11	1.0–53.6
ILTV	2021	8 (72.7)	11	46.4–99.9
IBV	2024	1 (5.0)	20	0.0–14.6
ILTV	2024	5 (25.0)	20	6.0–44.0

## Data Availability

The data presented in this study are available on request from the corresponding author.

## Abbreviations

The following abbreviations are used in this manuscript:

BPS	backyard production system
IBV	infectious bronchitis virus
ILTV	infectious laryngotracheitis virus
ELISA	Enzyme-Linked Immunosorbent Assay
qPCR	Quantitative Polymerase Chain Reaction
CI	confidence interval
COVID-19	Coronavirus Disease 2019

## References

[1] Mottet A., Tempio G. (2017). Global poultry production: Current state and future outlook and challenges. World’s Poult. Sci. J..

[2] OECD/FAO (2021). OECD-FAO Agricultural Outlook 2021–2030.

[3] Sonaiya E., Swan S. (2004). Small-Scale Poultry Production, Technical Guide Manual.

[4] Alders R.G., Campbell A., Costa R., Guèye E.F., Ahasanul Hoque M., Perezgrovas-Garza R., Rota A., Wingett K. (2021). Livestock across the world: Diverse animal species with complex roles in human societies and ecosystem services. Anim. Front..

[5] Diao X., Reardon T., Kennedy A., DeFries R.S., Koo J., Minten B., Takeshima H., Thornton P. (2023). The future of small farms: Innovations for inclusive transformation. Science and Innovations for Food Systems Transformation.

[6] von Braun J., Afsana K., Fresco L.O., Hassan M.H.A. (2023). Science and Innovations for Food Systems Transformation.

[7] Di Pillo F., Anríquez G., Alarcón P., Jimenez-Bluhm P., Galdames P., Nieto V., Schultz-Cherry S., Hamilton-West C. (2019). Backyard poultry production in Chile: Animal health management and contribution to food access in an upper middle-income country. Prev. Vet. Med..

[8] Hamilton-West C., Rojas H., Pinto J., Orozco J., Hervé-Claude L., Urcelay S. (2012). Characterization of backyard poultry production systems and disease risk in the central zone of Chile. Res. Vet. Sci..

[9] Baumberger C., Di Pillo F., Galdames P., Oyarzun C., Marambio V., Jimenez-Bluhm P., Hamilton-West C. (2023). Swine Backyard Production Systems in Central Chile: Characterizing Farm Structure, Animal Management, and Production Value Chain. Animals.

[10] Jimenez-Bluhm P., Di Pillo F., Bahl J., Osorio J., Schultz-Cherry S., Hamilton-West C. (2018). Circulation of influenza in backyard productive systems in central Chile and evidence of spillover from wild birds. Prev. Vet. Med..

[11] Bravo-Vasquez N., Baumberger C., Jimenez-Bluhm P., Di Pillo F., Lazo A., Sanhueza J., Schultz-Cherry S., Hamilton-West C. (2020). Risk factors and spatial relative risk assessment for influenza A virus in poultry and swine in backyard production systems of central Chile. Vet. Med. Sci..

[12] Bravo-Vasquez N., Di Pillo F., Lazo A., Jiménez-Bluhm P., Schultz-Cherry S., Hamilton-West C. (2016). Presence of influenza viruses in backyard poultry and swine in El Yali wetland, Chile. Prev. Vet. Med..

[13] Ayala A.J., Yabsley M.J., Hernandez S.M. (2020). A review of pathogen transmission at the backyard chicken–wild bird interface. Front. Vet. Sci..

[14] Jones P., Niemi J., Christensen J.-P., Tranter R., Bennett R. (2018). A review of the financial impact of production diseases in poultry production systems. Anim. Prod. Sci..

[15] Di Pillo F., Jimenez-Bluhm P., Baumberger C., Marambio V., Galdames P., Monti G., Schultz-Cherry S., Hamilton-West C. (2020). Movement Restriction and Increased Surveillance as Efficient Measures to Control the Spread of Highly Pathogenic Avian Influenza in Backyard Productive Systems in Central Chile. Front. Vet. Sci..

[16] Chaves Hernández A. (2014). Poultry and avian diseases. Encyclopedia of Agriculture and Food Systems.

[17] Laamiri N., Fällgren P., Zohari S., Ben Ali J., Ghram A., Leijon M., Hmila I. (2016). Accurate detection of avian respiratory viruses by use of multiplex PCR-based luminex suspension microarray assay. J. Clin. Microbiol..

[18] Hidalgo H. (2003). Infectious laryngotracheitis: A review. Braz. J. Poult. Sci..

[19] Ou S.-C., Giambrone J.J. (2012). Infectious laryngotracheitis virus in chickens. World J. Virol..

[20] Agunos A., Pierson F.W., Lungu B., Dunn P.A., Tablante N. (2016). Review of nonfoodborne zoonotic and potentially zoonotic poultry diseases. Avian Dis..

[21] Goraya M.U., Ali L., Younis I. (2017). Innate immune responses against avian respiratory viruses. Hosts Viruses.

[22] Brown Jordan A., Gongora V., Hartley D., Oura C. (2018). A review of eight high-priority, economically important viral pathogens of poultry within the Caribbean region. Vet. Sci..

[23] Seifi S., Asasi K., Mohammadi A. (2010). Natural co-infection caused by avian influenza H9 subtype and infectious bronchitis viruses in broiler chicken farms. Vet. Arh..

[24] Abdo W., Magouz A., El-Khayat F., Kamal T. (2017). Acute Outbreak of Co-Infection of Fowl Pox and Infectious Laryngotracheitis Viruses in Chicken in Egypt. Pak. Vet. J..

[25] ChileCarne (2021). La Industria en Cifras. Resumen de Cifras. https://www.chilecarne.cl/la-industria-en-cifras/.

[26] INE (2021). Censo Agropecuario y Forestal.

[27] Cubillos A., Ulloa J., Cubillos V., Cook J.K. (1991). Characterisation of strains of infectious bronchitis virus isolated in Chile. Avian Pathol..

[28] Lopez J.C., McFarlane R., Ulloa J. (2006). Detection and characterization of infectious bronchitis virus in Chile by RT-PCR and sequence analysis. Arch. Med. Vet..

[29] Guzmán M., Sáenz L., Hidalgo H. (2019). Molecular and Antigenic Characterization of GI-13 and GI-16 Avian Infectious Bronchitis Virus Isolated in Chile from 2009 to 2017 Regarding 4/91 Vaccine Introduction. Animals.

[30] Verdugo C., Pinto A., Ariyama N., Moroni M., Hernandez C. (2019). Molecular identification of avian viruses in neotropic cormorants (Phalacrocorax brasilianus) in Chile. J. Wildl. Dis..

[31] Gatica T., Salgado S., Reyes H., Loncoman C. (2025). Development of a qPCR Tool for Detection, Quantification, and Molecular Characterization of Infectious Laryngotracheitis Virus Variants in Chile from 2019 to 2023. Animals.

[32] Di Castri F., Hajek E.R. (1976). Bioclimatología de Chile.

[33] Aguayo M., Pauchard A., Azócar G., Parra O. (2009). Cambio del uso del suelo en el centro sur de Chile a fines del siglo XX: Entendiendo la dinámica espacial y temporal del paisaje. Rev. Chil. Hist. Nat..

[34] Di Rienzo J.A., Casanoves F., Balzarini M., Gonzalez L.A. *InfoStat*. [Programa de Cómputo] 2011; Versión 24-03-2011. http://www.infostat.com.ar/.

[35] Baumberger C., Anríquez G., Galdames P., Palma T., Gonzalez M.A., Orozco K., Oyarzun C., Rojas C., Marambio V., Ruiz S. (2025). Exposure Practices to Animal-Origin Influenza A Virus at the Animal–Human Interface in Poultry and Swine Backyard Farms. Zoonoses Public Health.

[36] Conan A., Goutard F.L., Sorn S., Vong S. (2012). Biosecurity measures for backyard poultry in developing countries: A systematic review. BMC Vet. Res..

[37] Iqbal M. (2009). Controlling avian influenza infections: The challenge of the backyard poultry. J. Mol. Genet. Med. Int. J. Biomed. Res..

[38] Bhuiyan M.S.A., Amin Z., Rodrigues K.F., Saallah S., Shaarani S.M., Sarker S., Siddiquee S. (2021). Infectious bronchitis virus (gammacoronavirus) in poultry farming: Vaccination, immune response and measures for mitigation. Vet. Sci..

[39] Gutierrez-Ruiz E., Ramirez-Cruz G., Camara Gamboa E., Alexander D., Gough R. (2000). A serological survey for avian infectious bronchitis virus and Newcastle disease virus antibodies in backyard (free-range) village chickens in Mexico. Trop. Anim. Health Prod..

[40] Pohjola L., Tammiranta N., Ek-Kommonen C., Soveri T., Hänninen M.-L., Fredriksson Ahomaa M., Huovilainen A. (2017). A survey for selected avian viral pathogens in backyard chicken farms in Finland. Avian Pathol..

[41] Chen G.-Q., Zhuang Q.-Y., Wang K.-C., Liu S., Shao J.-Z., Jiang W.-M., Hou G.-Y., Li J.-P., Yu J.-M., Li Y.-P. (2013). Identification and survey of a novel avian coronavirus in ducks. PLoS ONE.

[42] Hughes L.A., Savage C., Naylor C., Bennett M., Chantrey J., Jones R. (2009). Genetically diverse coronaviruses in wild bird populations of northern England. Emerg. Infect. Dis..

[43] Woo P., Lau S., Lam C., Lau C., Tsang A., Lau J., Bai R., Teng J., Tsang C., Wang M. (2012). Discovery of seven novel Mammalian and avian coronaviruses in the genus deltacoronavirus supports bat coronaviruses as the gene source of alphacoronavirus and betacoronavirus and avian coronaviruses as the gene source of gammacoronavirus and deltacoronavirus. J. Virol..

[44] Miłek J., Blicharz-Domańska K. (2018). Coronaviruses in avian species–review with focus on epidemiology and diagnosis in wild birds. J. Vet. Res..

